# Profiling Metabolites and Biological Activities of Sugarcane (*Saccharum officinarum* Linn.) Juice and its Product Molasses *via* a Multiplex Metabolomics Approach

**DOI:** 10.3390/molecules24050934

**Published:** 2019-03-07

**Authors:** Sara E. Ali, Rania A. El Gedaily, Andrei Mocan, Mohamed A. Farag, Hesham R. El-Seedi

**Affiliations:** 1 Department of Pharmaceutical Biology, Faculty of Pharmacy & Biotechnology, The German University in Cairo, New Cairo 12613, Egypt; saraezz16512@gmail.com; 2 Pharmacognosy Department, Faculty of Pharmacy, Cairo University, Kasr el Aini st., Cairo 11562, Egypt; rania.elgedaily@pharma.cu.edu.eg; 3 Department of Pharmaceutical Botany, “Iuliu Hațieganu” University of Medicine and Pharmacy, 400337 Cluj-Napoca, Romania; amocanm@gmail.com; 4 Department of Chemistry, School of Sciences & Engineering, The American University in Cairo, New Cairo 11835, Egypt; 5 Pharmacognosy Group, Department of Medicinal Chemistry, Uppsala University, Biomedical Centre, Box 574, SE-75 123 Uppsala, Sweden; hesham.el-seedi@ilk.uu.se; 6 Faculty of Science, Menoufia University, Shebin El-Kom 32512, Egypt

**Keywords:** *Saccharum officinarum*, molasses, UPLC/MS, GC/MS, antioxidant, metabolomics

## Abstract

Sugarcane (*Saccharum officinarum* L.) is an important perennial grass in the Poaceae family cultivated worldwide due to its economical and medicinal value. In this study, a combined approach using mass spectrometry (MS) and nuclear magnetic resonance (NMR) spectroscopy was employed for the large-scale metabolite profiling of sugarcane juice and its by-product molasses. The polyphenols were analysed via UPLC-UV-ESI-MS, whereas the primary metabolites such as sugars and organic and amino acids were profiled using NMR spectroscopy and gas chromatography/mass spectrometry (GC/MS). UPLC/MS was more effective than NMR spectroscopy or GC/MS for determining differences among the metabolite compositions of the products. Under the optimized conditions, UPLC/MS led to the identification of 42 metabolites, including nine flavonoids, nine fatty acids, and two sterols. C/O Flavone glycosides were the main subclass detected, with tricin-7-O-deoxyhexosyl glucuronide being detected in sugarcane and molasses for the first time. Based on GC/MS analysis, disaccharides were the predominant species in the sugarcane juice and molasses, with sucrose accounting for 66% and 59%, respectively, by mass of all identified metabolites. The phenolic profiles of sugarcane and molasses were further investigated in relation to their in vitro antioxidant activities using free radical scavenging assays such as 2,2-Diphenyl-1-picrylhydrazyl free radical-scavenging ability (DPPH), Trolox equivalent antioxidant capacity (TEAC) and ferric reducing antioxidant power (FRAP). In view of its higher total phenolic content (TPC) (196 ± 2.1 mg GAE/100 g extract) compared to that of sugarcane juice (93 ± 2.9 mg GAE/100 g extract), molasses exhibited a substantially higher antioxidant effect. Interestingly, both extracts were also found to inhibit α-glucosidase and α-amylase enzymes, suggesting a possible antihyperglycaemic effect. These findings suggest molasses may be a new source of natural antioxidants for functional foods.

## 1. Introduction

Sugarcane juice is well known as a raw material for the production of refined sugar, whereas its wax is a potential substitute for carnauba wax, which is used in cosmetics and pharmaceuticals but is rather expensive. In addition to refined sugar, a major product of sugarcane juice production, other by-products, including brown sugar, molasses, and jaggery, are made during processing [1]. Sugarcane juice contains a myriad of phytochemicals, including phenolics, sterols, terpenoids [2], lignins [3] and policosanols, which are major components of wax and plant oils [1]. The colour components from sugarcane juice are classified into four major classes: plant pigments, polyphenolic compounds, caramels, and degradation products from the condensation of sugars with amines [1]. Aside from its nutritive value, the biological effects attributed to the constituents of sugarcane include analgesic [4], antihyperglycaemic [5], diuretic [6], anti-inflammatory [7], antihypercholesterolemic [8] and antithrombotic effects [9].

The flavonoid content in sugarcane juice (0.6 mg/mL) was found to be comparable to the levels in other food resources, such as orange juice and black tea [10]. Various chromatographic techniques, such as liquid chromatography (LC) coupled to an ultraviolet (UV) diode array [10], LC coupled to an atmospheric pressure chemical ionization tandem mass spectrometry (APCI-MS) system [11], and LC coupled to electrospray ionization mass spectrometry (ESI-MS) [12], have been used to characterize the flavonoids in sugarcane leaves, juice, and bagasse. Several flavone glycosides, including orientin, tricin-7-*O*-neohesperidoside and tricin-7-*O*-glucoside, diosmetin-8-*C*-glycoside, schaftoside, isoschaftoside, vitexin, 4’,5’-dimethyl-luteolin-8-*C*-glycoside, luteolin 8-*C*-(rhamnosyl-glucoside), and flavolignan 7-*O*-glucosides along with their aglycones, have been identified.

Molasses is a viscous by-product of refining sugarcane into sugar through heating and is commonly used worldwide in human and animal feed as well as in ethanol production. Notably, phenolic acids, such as *p*-hydroxybenzoic acid, *p*-hydroxybenzaldehyde, vanillic acid, syringic acid and ferulic acid, are more abundant than other sugarcane products in molasses [13]. Dehydrodiconiferyl alcohol-9’-O-β-d-gluopyranoside and iso-orientin-7-3’-O-dimethylether were also isolated from molasses [14,15]. In addition, molasses has a high content of flavonoid derivatives and shows a higher antioxidant activity than is seen in other by-products [16].

Metabolomics can detect changes in food components that could occur during the food processing steps typical during molasses preparation [17]. While studies on sugarcane and molasses have targeted certain classes of metabolites in an attempt to provide comprehensive insight into the chemical composition of this economically important food, a large-scale metabolomics analysis was adopted in this study. Considering the diversity of metabolites typical in most food extracts, no single analytical platform can quantify and identify all metabolites within a sample. Thus, employing hyphenated techniques such as LC and gas chromatography (GC) coupled to MS in addition to nuclear magnetic resonance (NMR) spectroscopy is essential for obtaining the most complete profiles of the primary and secondary metabolites in a complex sample [18,19]. The goal of this study was to apply different metabolomics platforms to distinguish the chemical constituents of sugarcane juice and molasses by targeting both the primary and secondary metabolites. Furthermore, the phenolic profiles of sugarcane juice and molasses were further investigated in relation to their biological effects. Our work may be integrated to transcriptomic and proteomic data in future studies to select potential sugarcane by-products for production of novel compounds beyond that of sucrose.

## 2. Results and Discussion

**Figure 1:**
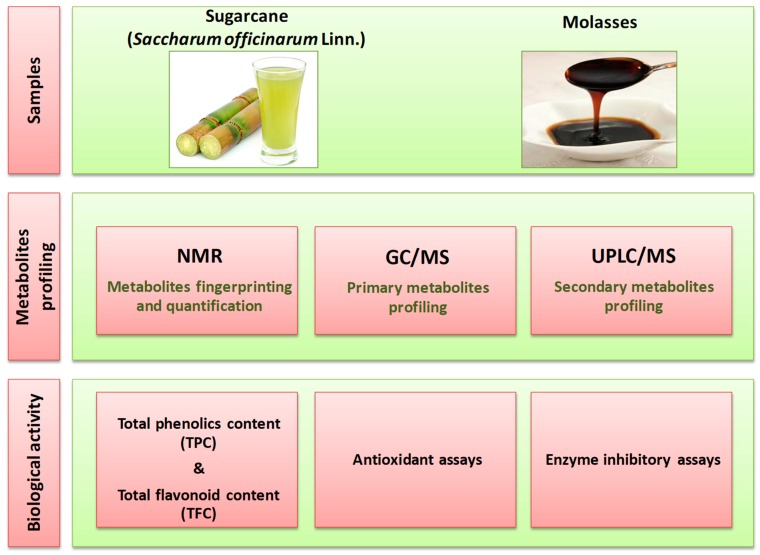
Schematic diagram for study experimental design.

The main goal of this study was to provide a comprehensive metabolite profile of sugarcane juice in comparison to its by-product, molasses. To achieve this goal, extracts were subjected to metabolite fingerprinting by NMR spectroscopy without a purification step, analogous to the procedures used for profiling via GC/MS and ultra-high-performance liquid chromatography (UPLC/MS). The combined use of NMR and MS analyses was a fundamental requirement to improve the coverage of metabolites and, moreover, facilitate identification. Furthermore, the total phenolic contents (TPC values) and total flavonoid contents (TFC values) of sugarcane juice and molasses were calculated and compared to biological assays including antioxidant activity assays and in vitro α-amylase and α-glucosidase inhibitory assays; see below for the experimental design (Figure 1).

### 2.1. H-NMR Spectroscopy Metabolite Fingerprinting and Quantification

**Figure 2:**
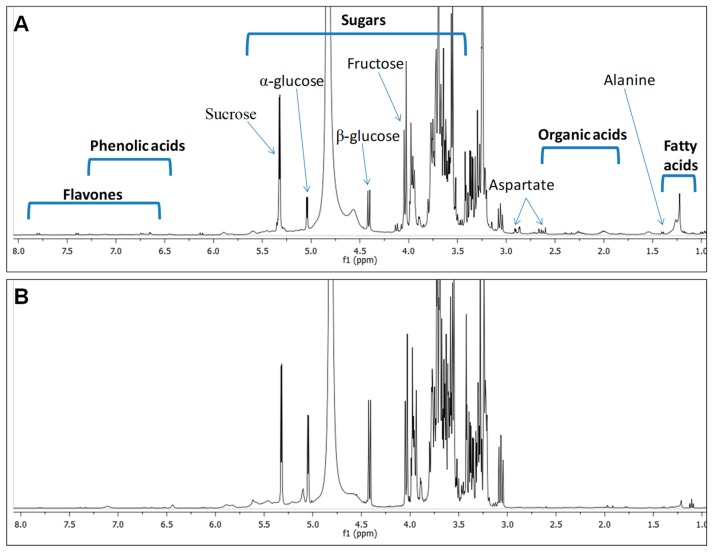
^1^H-NMR spectrum of sugarcane juice (**A**) and molasses (**B**). Identification of peaks is described in Table 1.

NMR spectroscopy for metabolite fingerprinting has been used to identify the major constituents present in sugarcane [20]. The advantages of high reproducibility along with its non-destructive and non-invasive nature make NMR spectroscopy well suited for plant metabolomics research [21,22]. In this study, ^1^H-NMR analysis led to the identification of 13 metabolites from sugarcane juice and molasses, and these compounds were identified as three amino acids, three organic acids, three sugars, one phenolic acid, two flavones, and one fatty acid. Most of these compounds were detected in both sugarcane juice and molasses but in different quantities. The identities, chemical shifts (δ values), coupling constants (*J* values) and multiplicities for all metabolites are presented in Table 1. Representative ^1^H-NMR spectra of sugarcane and molasses are shown in Figure 2.

The ^1^H-NMR spectra were divided into three main regions: a low-field region between 9.0 and 6.0 ppm in which the signals were mainly from the aromatic protons of flavonoid conjugates; a mid- to low-field region between 6.0 and 3.0 ppm with intense signals from the anomeric protons of sugar units; and a high-field region between 3.0 and 0.0 ppm in which the signals were from aliphatic protons of the fatty and organic acids. A detailed inspection of the ^1^H-NMR spectra revealed the abundance of sugars in both sugarcane and molasses, and clear signals characteristic of β-glucose (δ_H_ 4.42 ppm, d, *J* = 7.8 Hz), α-glucose (δ_H_ 5.12 ppm, d, *J* = 3.7 Hz), fructose (δ_H_ 4.04 ppm, d, *J* = 8.3 Hz) and sucrose (δ_H_ 5.39 ppm, d, *J* = 3.8 Hz) were observed. These assignments are in agreement with previous reports showing that the major metabolites in sugarcane are sugars [23]. Further quantitative NMR analyses were performed to characterize the major sugars, and the results are listed in Table 1.

The presence of amino acids in raw sugarcane juice and molasses is well established [24]. In this study, signals in the high-field region (3.0 and 0.0 ppm) indicated the presence of several amino acids, namely, valine (δ_H_ 1.00 ppm, d, *J* = 7.1 Hz), alanine (δ_H_ 1.41 ppm, d, *J* = 7.2 Hz), aspartate (δ_H_ 2.89, dd, *J* = 16.9, 3.4 Hz and δ_H_ 2.63, dd, *J* = 17.1, 9.5 Hz). These amino acids could originate from protein hydrolysis during the boiling stage of molasses production [24]. Furthermore, the organic acids in cane molasses include acids that naturally occur in cane juice, chiefly aconitic acid, in addition to those formed during the manufacture process, such as formic, acetic and lactic acids [25]. In the current study, the organic acids were identified based on the characteristic doublet peak appearing at (δ_H_ 1.33 ppm, d, *J* = 6.9 Hz) belonging to lactic acid (Table 1). The other organic acids identified include malic acid (δ_H_ 2.28, dd, J = 13.5, 1.7 Hz) and formic acid (δH 8.31 ppm, s). Although they constitute only a small fraction of the material, these acids are known for their buffering capacity, which allows them to control the pH of sugarcane juice.

A dihydroxybenzoic acid, gentisic acid, was identified based on its characteristic signals at δ_H_ 7.35 ppm (d, *J* = 3.6 Hz assigned to H-3), δ_H_ 6.75 ppm (dd, *J* = 8.5, 2.3 Hz assigned to H-5) and δ_H_ 6.67 ppm (d, *J* = 2.3 Hz assigned to H-6). Gentisic acid, previously reported as a metabolite in sugarcane [22], is analogous to salicylic acid in that it also functions as a signalling molecule in plant defence responses to pathogens and it might exhibit antimicrobial effects in molasses. The most abundant flavonoid compounds in *S. officinarum* are flavones (i.e., naringenin, apigenin, tricin and luteolin). These compounds the colour of sugarcane juice in addition to its health benefits due to their high antioxidant potential and capacity to protect cells from degenerative diseases [26]. Luteolin was identified in the low-field region from its characteristic signal at δ_H_ 6.73 ppm (d, *J* = 2.3 Hz assigned to H-6) and another proton doublet at δ_H_ 6.75 ppm (*J* = 2.3 Hz assigned to H-8). Furthermore, the presence of additional doublets at δ_H_ 7.41 ppm (*J* = 8.5 Hz assigned to CH-3’ & 5’), δ_H_ 7.80 ppm (*J* = 9.5 Hz assigned to CH-2’ & 6’) and δ_H_ 6.66 ppm (*J* = 2.3 Hz assigned to CH-6) indicated the presence of apigenin. These flavones are common flavonoids present in edible plants and functional foods used to treat a wide variety of diseases [22]. Aside from their physiological function *in planta*, these compounds exhibit antioxidant activities, chemopreventive effects and cytotoxic effects in humans [27].

Signals due to fatty acids appearing at δ_H_ 2.27 ppm (H-2), δ_H_ 1.60 ppm (H-3), δ_H_ 1.29 ppm ((CH_2_-)_n_ ω-2) and δ_H_ 1.32 ppm ((CH_2_-)_n_ ω-1) could not be attributed to an individual fatty acid. The NMR fingerprinting results are consistent with previous reports [22] indicating that this technique is not optimal for the comprehensive identification of discriminatory secondary metabolites in sugarcane owing to the predominated signals of sugars, which is typical of fruit juices. For quantification, fully resolved NMR signals specific for each unique metabolite were selected. The results are summarized in Table 1. Sugars are the major class of secondary metabolites quantified, with sucrose being the most abundant sugar, with concentrations of 126 and 67 µg/mg in molasses and sugarcane, respectively. Molasses was richer in flavones, with luteolin detected at 8.5 µg/mg versus 6.2 µg/mg in sugarcane. Apigenin, which was found at 11.2 µg/mg in molasses, was almost absent from sugarcane, as revealed via NMR spectroscopy. Phenolic acids such as gentisic acid were found at higher concentrations in molasses (10.6 µg/mg) than in sugarcane (6.5 µg/mg). High levels of aspartate amino acid (6.44 µg/mg) and lactic acid (1.54 µg/mg) were found in molasses, while malic acid (3.45 µg/mg) was more abundant in sugarcane.

### 2.2. Primary Metabolite Profiling of Sugars, Amino Acids and Organic Acids via GC/MS

**Figure 3:**
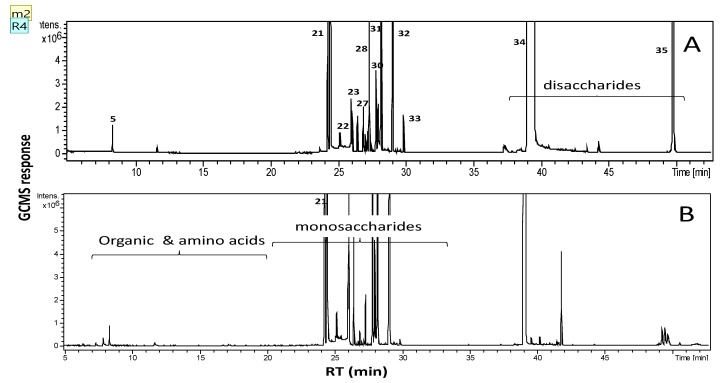
GC/MS chromatograms of silylated metabolites derived from sugarcane juice (**A**) and molasses (**B**) showing major peaks for sugars mono and disaccharides in both extracts.

Although ^1^H-NMR spectroscopy allowed the rapid and accurate quantification of the major metabolites in a single step, it only allows the detection of metabolites above a minimum threshold [28]. As a more sensitive technique, the metabolites present at low levels were profiled by GC/MS, which resulted in the detection of 32 metabolites (Table 2 and Figure 3). The metabolites included mono- and disaccharides as well as amino acids and organic acids. The identities, retention times (Rts) and retention indices (RI values) of the identified compounds are shown in Table 2. Disaccharides were found to be the most abundant compounds in sugarcane juice and molasses, with sucrose accounting 66% and 59%, respectively, of the identified metabolites (Table 2), which explains the intense, sweet taste of sugarcane [1]. However, the ratio of sucrose to apparent reducing sugars was dramatically lower in molasses; the mannose level increased to 10%.

Another sugar found at lower levels in sugarcane (5.6%) and molasses (7.9%) is d-tagatose, which has potential antidiabetic and anti-obesity effects. The proposed anti-obese mechanism involves interference with carbohydrate absorption by inhibiting intestinal disaccharidases and glucose transport or via hepatic inhibition of glycogenolysis [29]. Whether these effects can be observed following molasses intake has yet to be determined.

Significantly higher levels of rare sugars, i.e., d-allose, d -talose and d -psicose, were observed in molasses (3–6%) than in sugarcane juice (0.5–4%). d-Psicose exhibits antihyperglycaemic, antihyperlipidaemic, and anti-inflammatory effects [30,31], its zero-calorie intake and higher relative sweetness compared to sucrose make it attractive as a food ingredient for those wanting to lose weight and people with diabetes [32]. In general, based on the sugar profiles, molasses would provide a lower calorie intake relative to that associated with its juice.

Beyond being an established source of sugar [22], sugarcane is also known to contain organic acids, though at lower levels [25]. The higher levels of organic acids in molasses can likely be attributed to those that naturally occur in cane juice, such as malic acid and aconitic acid [25]. Acids, such as lactic acid (0.04%) and acetic acid (0.01%), can also be formed during the heating process. Aconitic acid, a tricarboxylic acid, is the most abundant acid in sugarcane [33].

### 2.3. Secondary Metabolites Profiling via UPLC/MS

MS-based metabolomics techniques provide an exceptional combination of sensitivity and selectivity, offering a powerful platform for a wide range of metabolomics studies. Moreover, the different MS methods provide an array of operational principles that can be applied, i.e., different ionization techniques, increasing the number of metabolites that can potentially be detected [28]. UPLC/MS is better suited for the analysis of secondary metabolites present at picomole to femtomole levels [34,35]. In the current study, UPLC coupled with high-resolution MS was employed to profile the secondary metabolites in sugarcane juice and molasses. LC–MS methods have been used to analyse sugarcane juice [12], and UPLC–Q-TOF-MS was selected in this study due to its superior peak resolution and higher sensitivity [18]. A total of 42 chromatographic peaks were detected, and of these, 27 were classified as *O*/*C* flavonoid glycosides, sterols and fatty acids. The metabolites were identified by comparing their retention times, UV-Vis spectra and MS data (accurate mass and fragmentation pattern). The chromatographic peaks and spectroscopic data are summarized in Table 3.

#### 2.3.1. Flavonoids

Sugarcane juice and molasses showed similar qualitative flavonoid profiles as exemplified by the presence of 1 di-*C*-glycoside (peak 6), 4 mono-*C*-glycosides (peaks 7, 8, 9, and 10), 2 *O*-glycosides (peaks 12, 13), one flavanone (peak 16) and one flavone (peak 17). The UV data suggested that most of the identified flavonoids were flavones, as they showed UV maxima at 271 and 332 nm. The next section presents the details of the identification of these major peaks.

Peak 6 exhibited a protonated molecular ion peak [M + H]^+^ at *m*/*z* 565.1544, and a fragmentation pattern typical of flavone-*C*-hexoside with fragments at *m*/*z* 547 corresponding to [M + H − 18]^+^, *m*/*z* 475 [M + H − 90]^+^, *m*/*z* 445 [M + H − 120]^+^, *m*/*z* 409 [M + H − 156]^+^, and *m*/*z* 379 [M + H − 186]^+^ was observed. This peak was assigned as schaftoside/isoschaftoside (apigenin-6-*C*-glucoside-8-*C*-arabinoside) [12]. Similarly, diosmetin-8-*C*-glucoside was identified as peak 7 based on its [M + H]^+^ signal at *m*/*z* 463.1234 and fragment ions at *m/z* 445 [M + H − 18]^+^, *m*/*z* 427 [M + H − 36]^+^, *m*/*z* 409 [M + H − 54]^+^, *m*/*z* 397 [M + H − 30–60]^+^, *m*/*z* 343 [M + H − 120]^+^, and *m*/*z* 313 [M + H − 150]^+^ [11]. Peak 8 exhibited an [M + H]^+^ ion at *m*/*z* 433.1133 and product ions at *m*/*z* 415 [M + H - 18]^+^, *m*/*z* 397 [M + H − 36]^+^, *m*/*z* 379 [M + H − 54]^+^, and *m*/*z* 313 [M + H − 120]^+^ and was therefore identified as swertisin (7-*O*-methylapegenin-6-*C*-glucoside) [36].

Peak 10, with an [M + H]^+^ ion at *m*/*z* 477.1395 showed the loss of 162 amu, corresponding to a hexose moiety linked through an *O*-linkage, to afford an aglycone of *m*/*z* 315 and was tentatively identified as dimethylluteolin-8-*O*-hexoside [37].

Peaks 12 and 13 exhibited molecular ions at *m*/*z* 653.1694 and 639.1903, respectively, and the fragment ion at *m*/*z* 331 for both compounds was attributed to tricin aglycone. The fragment ion at *m*/*z* 507 was indicative of the loss of glucuronic acid (−176 amu), while the loss of 308 amu from peak 13 indicated a deoxyhexosyl hexoside and led to these peaks being attributed to tricin-*O*-deoxyhexosyl glucuronide and tricin-*O*-neohesperidoside, respectively. This is the first report of tricin-*O*-deoxyhexosyl glucuronide in sugarcane and molasses. [11]. Additionally, this is the first report on the flavonoid profile of sugarcane-derived molasses.

#### 2.3.2. Fatty Acids and Sterols

Peaks 15, 18, 20, 22, 25, 31, 33, 36 and 37 were each attributed to a fatty acid and peaks 34 and 35 were attributed to sterols based on their predicted molecular formulas. Hydroxyoctadecadienoic acid ([M + H]^+^ at *m*/*z* 297.2424) was assigned as peak 31 and was found almost exclusively in molasses. Other hydroxylated fatty acids were assigned as peaks 15, 18 and 20; they showed masses of 311.2219, 272.2583 and 295.2261 amu and were tentatively assigned as epoxy-hydroxy-octadecadienoic acid, hydroxy palmitic acyl amide and hydroxydecatrienoic acid, respectively. The two sterol peaks (34 and 35) were tentatively assigned as tetrahydroxy cholanoic acid ([M + H]^+^ at *m*/*z* 425.2868) and its amide trihydroxycholanoyl amide (cholamide) ([M + H]^+^ at *m*/*z* 408.3082), and this is the first report of these compounds in *Saccharum* juice or molasses.

#### 2.3.3. Miscellaneous

Auxin (^1^H-indole-3-propenoic acid, peak 1) and 2 glyceryl conjugates (peaks 2/4 & 11), assigned as hydroxyphenylglycerol and hydroxyphenylglyceryl acetate, respectively, were found in sugarcane juice. In contrast, guaiacylglycerol-*O*-hexoside (peak 11) was found exclusively in molasses, which is consistent with previous reports on the isolation of several guaiacylglycerol derivatives from molasses [38].

### 2.4. TPC and TFC in Sugarcane Juice and Molasses

Antioxidants are widely used in the food and pharmaceutical industries owing to their ability to scavenge free radicals or interfere with their propagation [39]. With increasing interest in natural antioxidants, the antioxidant effects of sugarcane were assessed [40]. Sugarcane (*Saccharum officinarum* Linn.) is a rich source of natural antioxidants [26]. Hence, in view of its high content of phenolic compounds according to UPLC/MS analysis (Table 3), sugarcane may be a source of natural antioxidants [40]. The present study aimed to assess the TPC of sugarcane in comparison to that of molasses. As shown in Table 4, the TPC of molasses was higher (196 ± 2.1 mg GAE/100 g extract) than that of sugarcane juice (93 ± 2.9 mg GAE/100 g extract). This difference can be attributed to its high content of coloured components, as the colour of the material is highly dependent on phenolic compounds [15]. This result is in agreement with that of Iqbal et al. [41] revealing that molasses exhibited a higher TPC than other sugarcane by-products, such as jaggery, raw and refined sugar, and other reports [15] also showing that sugar products, particularly molasses, are a rich source of phenolic compounds. Previous studies [42] have shown that sugarcane is rich in flavonoid compounds, mainly in the form of C-glycosides [10]. In this study, the TFCs in sugarcane juice and molasses (Table 4) were comparable (259 mg QE/100 g extract and 297 mg QE/100 g, respectively). These results are in line with [10] that revealed flavonoid content in sugarcane to reach approximately 170 mg flavonoid/100 g of the fresh plant material. The high flavonoid contents in sugarcane juice and molasses make potential alternatives to other natural flavonoid resources [2], such as apples (98–143 mg flavonoid/100 g of fresh plant material) [43], onions (71–80) [44], tomatoes (0.5–3) [45] and honey (3) [10].

### 2.5. In vitro DPPH, ABTS and FRAP Antioxidant Assays of Sugarcane and Molasses

In the current study, the antioxidant capacities of sugarcane juice and molasses were further assessed using 2,2-Diphenyl-1-picrylhydrazyl free radical-scavenging ability (DPPH) and Trolox equivalent antioxidant capacity (TEAC) radical scavenging assays, which are widely used for estimating the free-radical scavenging activities of antioxidants from fruit and vegetable juices [2,46]. In agreement with [40], our data showed that sugarcane and molasses exhibited notable DPPH radical scavenging activities at value of 2.3 and 1.9 mg TE/g extract, respectively. Likewise, both extracts could scavenge 2,2′-azinobis-(3- ethylbenzothiazoline-6-sulfonic acid) (ABTS) radical cations at doses of 9.6 and 11.8 mg TE/g extract, respectively (Table 4). Their antioxidant activities can likely be attributed to the presence of flavonoid conjugates (i.e., apigenin, luteolin and tricin derivatives) [47] (Table 3).

Another antioxidant model, the ferric reducing antioxidant power (FRAP) assay, which is widely used to determine the antioxidant potentials of plant extracts based on their electron-donating abilities, was also investigated [2]. Similar to their radical scavenging rates, sugarcane and molasses showed appreciable antioxidant effects and were recorded to have a FRAP values of 13.46 ± 0.72 and 18.4 ± 0.9 mg TE/g extract, respectively (Table 4). The slightly higher antioxidant potential of molasses is consistent with its higher TPC (Table 4).

### 2.6. α-Amylase & α-Glucosidase Inhibition Assays

Controlling blood sugar levels through the inhibition of carbohydrate-metabolizing enzyme is a current approach for managing diabetes. α-Amylase is an enzyme responsible for the hydrolysis of complex starches into oligosaccharides, whereas α-glucosidase hydrolyses oligosaccharides into glucose and other monosaccharides.

Plant-based α-amylase and α-glucosidase inhibitors will likely be useful in regulating blood-glucose levels with a lower incidence of gastrointestinal side effects, as observed in acarbose [48]. In the current study, the α-amylase and α-glucosidase inhibitory activities of sugarcane and molasses were measured to assess their antihyperglycaemic potential, and we were encouraged by the presence of several sugars and flavonoids with potential hypoglycaemic effects, i.e., tagatose, psicose and luteolin (Table 1 and Table 2). As shown in Table 4, the α-amylase inhibitory activities of sugarcane and molasses were comparable at 0.5 ± 0.03 and 0.43 ± 0.04 mmol ACAE/g extract, respectively. In contrast, molasses exhibited a higher α-glucosidase inhibitory activity (6.2 ± 0.31 mmol ACAE/g extract) than sugarcane (3.61 ± 0.7 mmol ACAE/g extract) likely because of its considerably higher TPC (Table 4). Positive correlations between the TPC of a natural extract and its ability to inhibit glucosidase have been observed in previous reports [49]. The high flavonoids content (i.e., luteolin and apigenin) in sugarcane and molasses (Table 1 and Table 3) may contribute to their bioactivities. In fact, flavonoids (i.e., luteolin) exhibit α-glucosidase inhibitory effects comparable to that of acarbose. Luteolin also effectively inhibits α-amylase, although it is less potent than acarbose [50]. Another major compound found in molasses (Table 1) that is likely act synergistically with luteolin is gentisic acid, which is reported to exhibit α-amylase and α-glucosidase inhibitory activities [51]. These findings are in accordance with previous reports suggesting that the phenolic compound in sugarcane can effectively suppress postprandial hyperglycaemia in T2DM patients [52].

Taken together, our data reveal that the higher antioxidant and antihyperglycaemic activities observed in molasses, which are in good correlation with its high TPC and TFC values, are consistent with it having a more favourable sugar content compared to that of sugarcane juice. This study suggests that the phenolic fraction of molasses could be used as a new source of natural antioxidants for functional foods for patients with diabetes [16,52].

## 3. Materials and Methods

### 3.1. Extraction and Sample Preparation

Sugarcane juice was obtained from fresh *Saccharum officinarum* stems using a mechanical juicer. Until analysis, the obtained juice was kept at −80 °C to avoid autooxidation. Molasses was manufactured and provided by El-Bawady Co., 6th October, Egypt. Both sugarcane juice and molasses were lyophilized overnight to remove water, and their nutritive values were assessed on a solid basis. Three biological replicates were analysed for each sample.

### 3.2. Chemicals and Reagents

Methanol-*d*_4_ (99.80% D) and trimethylsilyl propionate (TSP) were purchased from Deutero GmbH (Kastellaun, Germany). Acetonitrile (HPLC grade) and formic acid (LC–MS grade) were obtained from J. T. Baker (Deventer, The Netherlands). Chromabond C_18_ solid phase extraction (SPE) cartridges (500 mg stationary phase, 3 mL) were purchased from Macherey & Nagel (Düren, Germany). Flavonoid standards were purchased from Indofine (Somerville, NJ, USA). All other chemicals were provided by Sigma-Aldrich (St. Louis, MO, USA).

### 3.3. NMR Analysis of Sugarcane Juice and Molasses

The lyophilized extracts of the juice and molasses (120 mg) were each homogenized with 5 mL of 100% methanol for 5 min. A 3-mL aliquot of this mixture was prepared for NMR analysis by evaporation of the solvent under a nitrogen stream and reconstituting the dried pellet in 800 µL of methanol-*d*4. After centrifugation (1 min, 13000 g), supernatants were transferred to NMR tubes (5 mm). All spectra were recorded on an Agilent VNMRS 600 NMR spectrometer (Agilent Technologies Inc., Santa Clara, CA, USA); the operating conditions are as described in [53]. The 1D ^1^H-NMR spectra were acquired under quantitative conditions using a 90° single-pulse experiment. The spectral width and the acquisition time were set to 12,500 Hz, and frequency tuned and impedance matched before each sample run. The receiver gain was set to 45.2 for all ^1^H-NMR measurements. A total of 160 transients were acquired with a relaxation delay of 24 s, which is more than 5 times the longest T1 observed for TSP. For the quantification to be optimal, full relaxation of the protons of targeted NMR signals and the internal standard has to be achieved. For this purpose a rather large sum of relaxation delay and acquisition time of 25 s was used, as the longest relaxation times were 4.5 s for the TSP protons. Zero filling up to 128 K and an exponential window function with lb = 0.4 was used prior to Fourier transformation.

2D NMR spectra were recorded using standard CHEMPACK 6.2 pulse sequences (gDQCOSY, gHSQCAD, gHMBCAD) implemented in standard VNMRJ 4.0A spectrometer software. The HSQC experiment was optimized for 1JCH = 146 Hz with DEPT-like editing and 13C-decoupling during acquisition time, sw = 7183.9 Hz; sw1 = 34692.1, nt = 16, ni = 200 Hz per Point: 6.7 in F2; 173.0 in F1, delay adjusted to nJ (CH) = 8 Hz at 62.5 ms. The HMBC experiment was optimized for a long-range coupling of 8 Hz; a 2-step 1JCH filter was used (130–165 Hz), sw = 7183.9 Hz; sw1 = 34692.1, nt = 16, ni = 200 Hz per Point: 6.7 in F2; 173.0 in F1, relevant delay adjusted to nJ (CH) = 8 Hz at 62.5 ms. The COSY experiment was optimized for a spectral width in both dimensions of 7183.9 Hz, nt = 4, ni = 256, resolution set at 6.7 in F2 & 28.0 in F1 Hz per point, respectively.

### 3.4. NMR Quantification

For NMR quantification and calibration of the chemical shifts, TSP was added to a final concentration of 100 µM. The ^1^H-NMR spectra were automatically Fourier transformed to ESP files using ACD/NMR Manager lab version 10.0 software (Toronto, ON, Canada). The spectra were referenced to the internal standard (TSP) and processed as described in [54].

To quantify the metabolites of sugarcane and molasses using NMR spectroscopy, the peak areas of the selected proton signals belonging to the target compounds and the peak area of TSP were manually integrated for all the samples. The following equation was then used. m_T_ = mass of the target compound in the solution used for the ^1^H-NMR analysis [μg]:

(1) $$m_{T}=\;M_{T}\;\times \;\frac{I_{T}}{I_{\mathrm{St}}}\times \;\frac{x_{\mathrm{St}}}{x_{T}}\times c_{\mathrm{St}}\times v_{\mathrm{St}}$$

M_T_—molecular weight of the target compound [g/mol];
I_T_—relative integral value of the ^1^H-NMR signal of the target compound;
I_St_—relative integral value of the ^1^H-NMR signal of the standard compound;
x_St_—number of protons belonging to the ^1^H-NMR signal of the standard compound;
x_T_—number of protons belonging to the ^1^H-NMR signal of the target compound;
c_St_—concentration of the standard compound in the solution used for ^1^H-NMR analysis [mmol/L];
v_St_—volume of the solution used for ^1^H NMR analysis [mL].

The analytical method was validated by determination of the selectivity and stability, according to our previous report [54]. The selectivity was assessed by visual comparison between ^1^H-NMR spectra of *S. officinarum* with and without internal standard. Moreover two-dimensional ^1^H–1H COSY, HMBC and HSQC spectra were also applied to reveal possible hidden signals. The precision tests were performed by repeating measurements of the same sample after 24 h with relative standard deviation (RSD) values considered as a measure of precision.

### 3.5. GC/MS Analysis of Silylated Primary Metabolites in Molasses and Sugarcane Juice

To analyse the primary metabolites (amino acids, organic acids, and sugars), 100 μL of the 50% aqueous extract (prepared by extracting 100 mg of dried molasses and sugarcane residues with 5 mL of 50% MeOH) was concentrated under nitrogen to complete dryness. For derivatization, 150 μL of N-methyl-N-(trimethylsilyl)-trifluoroacetamide (MSTFA) was then added, and the mixture was incubated at 60 °C for 45 min. The samples were analysed using GC/MS. The silylated derivatives were separated on a Rtx-5MS column (30 m length, 0.25 mm inner diameter, and 0.25 μm film thickness). Injections were made in split mode (1:15), and the remaining conditions were as follows: injector 280 °C, column oven 80 °C for 2 min, heating at a rate 5 °C/min to 315 °C, held at 315 °C for 12 min. He as the carrier gas at 1 mL.min^−1^. The transfer line and ion source temperatures were set at 280 and 180 °C, respectively.

The metabolites were identified via GC/MS using the procedure described by Farag et al. [54]. Briefly, the raw data acquired from Xcalibur 1.4 (Thermo Fisher Scientific, Inc., Waltham, MA, USA) were exported in NetCDF format using the File Converter tool in Xcalibur software. An automated mass spectral deconvolution and identification system (AMDIS 2.64, NIST, Gaithersburg, MD, USA, www.amdis.net) was used to deconvolute the acquired mass spectra prior to the database search. The RI values were calculated relative to a standard *n*-alkane mixture (C_8_-C_40_). The spectra of the individual components were transferred to the NIST Mass Spectral Search Program MS Search 2.0. The metabolites were identified by matching their mass spectra to reference spectra in the NIST Mass Spectral Library 2005 (National Institute of Standardization and Technology, Gaithersburg, MD, USA) and Golm Metabolome Database (Golm.mpg.de/csbdb/gmd/home/gmd_sm.html).

### 3.6. RP-UPLC/MS of the Secondary Metabolites in Molasses and Sugarcane

Fifteen milligrams of the dried lyophilized extracts of sugarcane juice and molasses were reconstituted in 1000 µL of 100% methanol and then filtered through a 22-µm filter. A 2-µL aliquot of the filtrate was injected in the UPLC system (Dionex UltiMate 3000, Thermo Fisher Scientific), which was equipped with a photodiode array detector (PDA, 220–600 nm) and a Waters Acquity HSS T3 RP column (150 × 1 mm, 1.8 µm particle size, pore size of 0.22 µm). Eluents A and B were water and acetonitrile, respectively, both containing 0.1% (*v*/*v*) formic acid. After a 1 min isocratic step at 5% eluent B, the samples were eluted with a linear gradient to 100% eluent B in 10 min at a flow rate of 150 µL/min. The column was washed with acetonitrile for 8 min and equilibrated for 11 min. The eluate from the PDA detector was transferred to an on-line hybrid LTQ-Orbitrap Elite mass spectrometer (Thermo Fisher Scientific, Darmstadt, Germany) equipped with a heated electrospray ion source (3 kV/4 kV ion spray voltage in the negative/positive ion mode, 300 °C capillary temperature, 250 °C source temperature, with nitrogen as the sheath and auxiliary gas). The MS spectra were acquired over the *m*/*z* range of 100–1500 at a resolution of 30,000. The collision-induced dissociation (CID) mass spectra were acquired in an ion trap with an activation frequency of 250 and an activation time of 10 ms using He as the collision gas. The metabolites were identified by their characteristic retention times (R_t_ values) and *m*/*z* values. The annotated metabolites were identified by MS/MS spectral similarity based on the phytochemical dictionary of the natural products database (CRC, Wiley) and an in-house spectral library. UPLC/MS validation was previously reported [19], with recovery percentages for kaempferol added prior to the extraction of samples was 96% with a limit of detection (LOD) and limit of quantification (LOQ) of 1.02 and 3.42 lg/ml, respectively.

### 3.7. TPC

The TPC values were determined using the Folin–Ciocâlteu method described by Mocan et al. [55]. For the high-throughput analysis of the samples, a SPECTROstar Nano Multi—Detection Microplate Reader with 96-well plates (BMG Labtech, Ortenberg, Germany) was used. Briefly, a mixture of 20 µL of the extract, 100 µL of Folin–Ciocâlteu reagent and 80 µL of sodium carbonate (Na_2_CO_3_, 7.5% *w*/*v*) was homogenized and incubated at room temperature in the dark for 30 min. Afterwards, the absorbance of the sample was measured at 760 nm. Gallic acid was used as a reference standard, and the TPC is expressed as gallic acid equivalents (GAE) in mg/g dry weight (dw) of plant material.

### 3.8. TFC

The TFC values were calculated and expressed as quercetin equivalents using a previously described method [56]. Briefly, a 100-µL aliquot of 2% AlCl_3_ aqueous solution was mixed with 100 µL of the sample. After an incubation time of 15 min, the absorbance of the sample was measured at 420 nm. Quercetin was used as a reference standard, and the TFC is expressed as quercetin equivalents (QE) in mg/g dry weight (dw) of plant material.

### 3.9. DPPH Radical Scavenging Assay

This assay was performed using a SPECTROstar Nano microplate reader (BMG Labtech, Offenburg, Germany). Each of the 96 wells contained 30 µL of sample solution (at an appropriate dilution) and 0.004% methanolic DPPH solution. The mixture was incubated for 30 min in the dark, and the reduction of the DPPH radical was determined by measuring the absorption of the sample at 515 nm. Trolox was used as a standard reference, and the results are expressed as Trolox equivalents per g dry weight of herbal extract (mmolTE/g dw herbal extract) [57].

### 3.10. TEAC Assay

The radical scavenging activity against the stable synthetic ABTS radical cation was measured using the method previously described by [55]. A Trolox calibration curve was prepared by plotting the percentage of ABTS radicals scavenged as a function of concentration. The final results are expressed as milligrams of Trolox equivalents (TE) per gram of dry herbal extract (mgTE/g dw extract).

### 3.11. FRAP Antioxidant Capacity Assay

In the FRAP assay, the reduction of Fe^3+^-TPTZ to the blue-coloured Fe^2+^-TPTZ complex was monitored by the method described by [58] with slight modifications. The FRAP reagent was prepared by mixing ten volumes of acetate buffer (300 mM, pH 3.6), one volume of TPTZ solution (10 mM TPTZ in 40 mM HCl) and one volume of FeCl_3_ solution (20 mM FeCl_3_•6H_2_O in 40 mM HCl). The reaction mixture (25 µL of sample and 175 µL of FRAP reagent) was incubated in the dark for 30 min at room temperature, and the absorbance of each solution was measured at 593 nm using a SPECTROstar Nano Multi-Detection Microplate Reader with 96-well plates (BMG Labtech). A Trolox^TM^ calibration curve was prepared by plotting the formation of the blue-coloured Fe^2+^-TPTZ complex as a function of concentration, and the results are expressed as milligrams of Trolox equivalents (TE) per milligram of extract (mg TE/mg extract).

### 3.12. α-Amylase Inhibition Assay

The α-amylase inhibitory activity was assessed using the method previously described by [59] with slight modifications. A 25-μL aliquot of sample solution was mixed with 50 μL of α-amylase solution (porcine pancreas, Sigma) in phosphate buffer (pH 6.9 with 6 mM sodium chloride) in a 96-well microplate and incubated for 10 min at 37 °C. After this preincubation period, the reaction was initiated by the addition of 50 μL of starch solution (0.05%). The reaction mixture was incubated for an additional 10 min at 37 °C and then stopped with the addition of 25 μL of HCl (1 M). Next, 100 μL of iodine–potassium iodide solution was added, and the absorbance was recorded at 630 nm. The α-amylase inhibitory activity is expressed as millimoles of acarbose equivalents per gram of dry herbal extract (mmol ACAE/g extract dw).

### 3.13. α-Glucosidase Inhibition Assay

The α-glucosidase inhibitory activity was tested using the protocol previously described by [59] with slight modifications. A 50-μL aliquot of sample solution was mixed with 50 μL of glutathione (0.5 mg/mL), 50 μL of α-glucosidase solution (from *Saccharomyces cerevisiae*, Sigma-Aldrich, Darmstadt, Germany) in phosphate buffer (pH 6.8), and 50 μL of 10 mM PNPG (*p*-nitrophenyl-β-d-glucuronide) (Sigma-Aldrich) solution in a 96-well microplate and incubated for 15 min at 37 °C. The reaction was stopped with the addition of 50 μL of 0.2 M sodium carbonate. The absorbance was recorded at 400 nm, and the α-glucosidase inhibitory activity is expressed as millimoles of acarbose equivalents per gram of dry herbal extract (mmol ACAE/g extract dw).

## 4. Conclusions

The conversion of sugarcane into economically viable food products is one of the main challenges in the sugarcane industry. In this study, a large-scale metabolite profiling of sugarcane and its by-product molasses was performed using various analytical platforms. UPLC/MS was found to be more effective than NMR and GC/MS for elucidating the differences in the composition of secondary metabolites as it provides better coverage of different metabolite classes, such as flavonoids and phenolic acids. The phenolic profiles of sugarcane and molasses was further compared in relation to their in vitro antioxidant activities using free-radical scavenging assays, namely, DPPH, TEAC and FRAP assays. In view of its higher TPC, the antioxidant activity of molasses was substantially higher than that of sugarcane (Table 4). Interestingly, the extracts were also able to inhibit enzymes such as α-glucosidase and α-amylase; however, their abilities to prevent increases in post-prandial glucose levels have not yet been determined. The data reported herein emphasize the potential health benefits of molasses and suggest the use of its phenolic fraction in functional foods for diabetic patients owing to its antioxidant and anti-hyperglycaemic activities. These results are particularly important in the food industry, as they highlight the importance of sugarcane as an inexpensive yet very nutritious consumable commodity. In addition, based on sugar profiling via GC/MS and NMR, molasses should provide a lower calorie intake compared to its juice. The determination of the inhibitory activity of sugarcane extract against other carbohydrate-degrading enzymes such as sucrase and isomaltase should now follow to further clarify its effects on sugar metabolism in animals.

## Figures and Tables

**Table 1:** Resonance assignments with chemical shifts of constituents identified molasses and sugarcane juice (methanol-*d4*) 600 MHz with concentration of metabolites represented in µg/mg (*n* = 2).

Class	Metabolite	δ^1^H (ppm) and Multiplicity	Assignments	Molasses	Sugarcane
**Amino acids**	Valine	1.00 (d, *J* = 7.1 Hz)	CH_3_-4,5	0.87	0.61
	Alanine	1.41 (d, *J* = 7.2 Hz); 3.23 (d, *J* = 1.7 Hz)	CH_3_-3; CH-2	1.67	1.0
	Aspartate	2.89 (dd, *J* = 16.9, 3.4 Hz); 2.63 (dd, *J* = 17.1, 9.5 Hz)	CH_2_-3	-	6.44
**Organic acids**	Lactic acid	1.33 (d, *J* = 6.9 Hz)	CH_3_-3	1.54	-
	Malic acid	2.28 (dd, *J* = 13.5, 1.7 Hz)	CH_2_-3	-	3.45
	Formic acid	8.31 (s)	HCOOH	0.94	0.4
**Sugars**	β-Glucose	4.42 (d, *J* = 7.8 Hz); 3.11 (d, *J* = 1.8 Hz); 3.36 (m); 3.22 (d, *J* = 2.0 Hz); 3.24 (m)	CH-1; CH-2; CH-3; CH-4; CH-5	57.1	11.2
	α-Glucose	5.12 (d, *J* = 3.7 Hz); 3.61 (d, *J* = 2.9 Hz)	CH-1; CH-5	51.1	10.2
	Fructose	4.04 (d, *J* = 8.3 Hz); 3.56 (m)	CH-5 (α-Fructofuranose); CH_2_-6 (β-Fructofuranose)	+	+
	Sucrose	5.39 (d, *J* = 3.8 Hz); 4.11 (d, *J* = 8.3 Hz)	CH-1 (glucose)CH-3 (fructose)	126	67
**Phenolic acids**	Gentisic acid	7.35 (d, *J* = 3.6 Hz); 6.75 (dd, *J* = 8.5, 2.3 Hz); 6.67 (d, *J* = 2.3 Hz)	CH-3; CH-5; CH-6	10.6	6.5
**Fatty acids**	Fatty acids	2.27 (m); 1.60 (m); 1.29 (m); 1.32 (m)	H-2; H-3; (CH_2_-)_n_ ω-2; (CH_2_-)_n_ ω-1	+	+
**Flavones**	Luteolin	6.73 (d, *J* = 2.3 Hz); 6.75 (d, *J* = 2.3 Hz)	CH-6; CH-8	8.5	6.2
	Apigenin	7.41 (d, *J* = 8.5 Hz); 7.80 (d, *J* = 9.5 Hz); 6.66 (d, *J* = 2.3 Hz)	CH-3’ & 5’; CH-2’ & 6’; CH-6	11.2	-

s: singlet; d: doublet; dd: double doublet; t: triplet; m: multiplet; +: present; -: absent.

**Table 2:** The identity, retention time (Rt), retention index (RI) and percentile levels (%) of primary silylated metabolites in sugarcane juice and molasses via GC/MS analysis (*n* = 2).

Name	Class	Rt (min)	RI	Sugarcane (%)	Molasses (%)
Ethylene glycol (2TMS)	Alcohol	7.883	1122	0.34	0.67
Phosphate (3TMS)	Inorganic acid	11.569	1269	0.22	0.51
Pipecolic acid (*N*,*O*-2 TMS)	Nitrogenous compound	17.857	1517	0.03	0.08
Phenylethanolamine (3TMS)		20.473	1629	0.01	0.01
*N*,*O*-hydroxylamine (2TMS)		8.346	1141	0.55	1.23
Aminoethanol, *O*,*N*,*N*-tris-TMS		18.022	1525	0.02	-
Lactic acid (2TMS)	Organic acid	6.394	1055	0.01	0.04
Acetic acid (TMS)		6.782	1073	-	0.01
Glyceric acid (3TMS)		13.103	1329	0.02	0.03
Glyceric acid isomer (3TMS)		13.738	1353	0.02	0.04
Acetoacetic acid (2TMS)		16.671	1470	0.01	0.1
Malic acid (3TMS)		17.078	1486	0.09	0.21
Itaconic acid (2TMS)		19.971	1607	0.01	0.03
trans-Aconitic acid (3TMS)		23.098	1747	0.07	0.12
Citric acid (4TMS)		24.523	1813	0.07	0.01
α-d-Ribofuranoside, methyl (3TMS)	Sugars	17.549	1505	-	0.01
Threonic acid (4TMS)		18.368	1539	0.01	0.01
Gluconic acid, 2-methoxime (4TMS)		20.804	1644	-	0.01
Ribitol (5TMS)		22.381	1714	0.01	0.02
Arabinofuranose (4TMS)		23.605	1771	0.06	0.04
Tagatofuranose (5TMS) (isomer 2)		24.216	1798	5.58	7.92
Mannopyranose, 6-deoxy-(4TMS)		25.093	1841	0.20	1.05
Psicose (5TMS)		25.927	1881	0.52	3.14
Mannopyranose (5TMS)		26.069	1888	2.74	9.19
Glucitol (6TMS)		26.721	1921	0.02	0.13
Fructose oxime (6TMS)		26.966	1933	0.06	0.09
Fructose (5TMS)		27.123	1941	0.72	0.89
Talose (5TMS)		27.772	1975	4.28	5.94
Allopyranose (5TMS)		27.786	1975	4.28	5.94
Galactofuranose (5 TMS)		27.901	1981	1.44	1.65
Gluconic acid δ-lactone (4TMS)		28.143	1993	1.49	-
Glucopyranose (5TMS)		28.97	2035	6.30	-
Inositol (6TMS) myo-		29.77	2076	0.26	-
Sucrose (8TMS)		38.866	2539	60.69	58.8
Sucrose (8TMS)		49.563	3083	5.25	-
Unknown	unknown	24.402	1808	4.62	2.120
Total				100.00	100.00

**Table 3:** Metabolite assignments for peaks tentatively identified in UPLC-UV-ESI-MS chromatograms of molasses and sugarcane juice in positive ionization mode.

Peak No.	M + H	Rt. (sec.)	UV (nm)	Fragments MS/MS^+^	Formula	Er. ppm	Identification	Class	Scj.	M
1	188.0705	144	220	163	C_11_H_10_NO_2_^+^	0.3	^1^H-Indole-3-propenoic acid	A	+	-
2	185.0801	186	221	163, 158, 141	C_9_H_13_O_4_^+^	8.1	Hydroxyphenyl)glycerol	GD	+	-
3	257.1013	198	221	222, 195, 185, 163, 145	C_12_H_17_O_6_^+^	2.7	Unknown		-	+
4	227.0914	204	221,301	223, 215, 195, 185	C_11_H_15_O_5_^+^	0.1	Hydroxyphenyl)glyceryl acetate	GD	+	-
5	249.0737	222	221	227, 209, 185, 163, 141	C_13_H_13_O_5_^+^	8.4	Unknown		+	-
6	565.1544	246	223,272,334	547, 475, 445, 409, 379	C_26_H_29_O_14_^+^	1.5	Schaftoside/ Isoschaftoside	Fl	+	+
7	463.1234	252	223,271,345	445, 427, 409, 397, 343, 313	C_22_H_23_O_11_^+^	0.1	Diosmetin-8-*C*-glucoside	Fl	+	+
8	433.1133	270	223,271,336	415, 397, 379, 367, 337, 313	C_21_H_21_O_10_^+^	0.8	Vitexin	Fl	+	+
9	447.1294	276	222,272,332	297, 327	C_22_H_23_O_10_^+^	1.8	Swertisin	Fl	+	+
10	477.1395	288	262,345	459, 441, 423, 411, 381, 357, 327, 315	C_23_H_25_O_11_^+^	0.7	4’, 5’Dimethylluteolin-8-*C*-glucoside.	Fl	+	+
11	377.1431	294	280		C_16_H_25_O_10_^+^	3.0	Guaiacylglycerol-*O*-hexoside	GD	-	+
12	653.1694	306	224,351	507, 331	C_29_H_33_O_17_^+^	2.7	Tricin7-*O*-deoxyhexosyl-glucuronide	Fl	+	+
13	639.1903	312	222,346	331	C_29_H_35_O_16_^+^	2.6	Tricin-7-*O*-neohesperidoside	Fl	+	+
14	353.23	444	223	325, 313, 303, 295, 277, 233, 203, 194	C_20_H_33_O_5_^+^	6.4	Unknown diterpene	DT	+	+
15	311.2219	492	223	295, 254, 213, 202	C_18_H_31_O_4_^+^	0.8	Epoxy-hydroxy-octadecadienoic acid	HFA	+	-
16	257.0792	504	223	230, 175	C_15_H_13_O_4_^+^	6.5	Liquiritigenin/ isoliquiritigenin	Fl	+	+
17	271.0605	522	223	257, 236, 226	C_15_H_11_O_5_^+^	1.4	Apigenin	Fl	-	+
18	272.2583	528	223	251, 226,209	C_16_H_34_NO_2_^+^	0.4	Hydroxy palmitic acyl amide	HFAA	+	-
19	381.2608	570	224	365, 341, 325, 303	C_22_H_37_O_5_^+^	7.2	Unknown diterpene	DT	+	-
20	295.2261	576	224	285, 277, 259, 249	C_18_H_31_O_3_	2.3	Hydroxy-octadecatrienoic acid	HFA	+	-
21	345.2429	594	224	337, 325, 297, 285, 251, 226	C_22_H_33_O_3_^+^	1.3	Unknown diterpene	DT	-	+
22	297.2424	600	224	277, 197	C_18_H_33_O_3_^+^	0.1	Hydroxy-octadecadienoic acid	HFA	-	+
23	335.2197	630	224	325, 303, 295, 285	C_20_H_31_O_4_^+^	5.8	Unknown diterpene	DT	+	-
24	335.219	678	224	325, 319, 313, 303, 295, 285, 249	C_20_H_31_O_4_^+^	8.0	Unknown diterpene	DT	-	+
25	279.231	684	224	269, 249, 235, 226, 197	C_18_H_31_O_2_^+^	3.1	Octadecatrienoic acid	FA	+	-
26	411.0939	738	224	389, 371, 365, 345, 331, 325, 317, 301	C_29_H_15_O_3_^+^	8.6	Unknown		-	+
27	341.2662	792	225	319, 297, 275, 253	C_20_H_37_O_4_^+^	7.0	Unknown diterpene	DT	-	+
28	297.2398	792	225	285, 275, 267, 253	C_18_H_33_O_3_^+^	9.0	Hydroxy Octadecatrienoic acid	HFA	+	-
29	355.2829	822	225	365, 355, 337, 325, 301, 279, 263, 245, 197	C_21_H_39_O_4_^+^	3.9	Octadecadienoyl glycerol	GD	-	+
30	439.3556	834	225	399, 385, 355, 341, 325, 311, 301, 285	C_30_H_47_O_2_^+^	3.4	Unknown triterpene	TT	-	+
31	277.2141	864	225	267, 261, 255, 243, 237	C_18_H_29_O_2_^+^	7.6	Octadecatetraenoic acid	FA	+	-
32	353.2661	870	225	331,325,313, 301	C_21_H_37_O_4_^+^	7.1	Unknown		+	-
33	281.2471	876	225	281, 263, 245, 226	C_18_H_33_O_2_^+^	1.5	Linoleic acid	FA	+	+
34	425.2868	888	226	365, 301, 285, 267, 241, 226.	C_24_H_41_O_6_^+^	6.9	Tetrahydroxycholanoic acid	St		
35	408.3082	912	226	365, 325, 301, 282, 266, 250	C_24_H_42_NO_4_^+^	6.4	Cholamide	ST	+	+
36	257.2464	918	226	239, 226	C_16_H_33_O_2_^+^	4.3	Palmitic acid	FA	+	+
37	283.2638	936	226	265,257, 226	C_18_H_35_O_2_^+^	2.3	Oleic acid	FA	+	-
38	397.3292	1008	227	375, 365, 355, 325, 312, 301	C_24_H_45_O_4_^+^	5.2	Unknown fatty acid	FA	+	+
39	338.342	1014	227	321, 301, 259, 226	C_22_H_44_NO^+^	0.6	Docosenoic acyl amide	NAA	+	+
40	413.2665	1020	227	393, 365, 338	C_26_H_37_O_4_^+^	5.2	Unknown fatty acid	FA	+	+
41	353.3029	1026		331, 315, 301	C_22_H_41_O_3_^+^	6.1	Unknown		+	+
42	469.3861	1068		430, 413, 365, 301	C_28_H_53_O_5_^+^	5.6	Unknown		-	+

A: auxin, DT: diterpene, FA: fatty acid, Fl: flavonoid, GD: glycerol derivative, HFA: hydroxy fatty acid, M: molasses, Naa: nacylamide, Scj.:sugarcane juice, ST: sterol and TT: triterpene.

**Table 4:** Total phenolics and flavonoids content, antioxidant capacity assays and α-amylase and α-glucosidase inhibitory capacities of sugarcane juice and molasses.

	Total Phenolics and Flavonoids Content		Antioxidant Capacity Assays			Enzyme Inhibitory Capacities	
	TPC (mg GAE/100g Extract)	TFC (mg QE/100g Extract)	DPPH Scavenging (mgTE/g Extract)	ABTS Scavenging (mgTE/g Extract)	FRAP (mgTE/g Extract)	α-Amylase Inhibition (mmolACAE/g Extract)	α-Glucosidase Inhibiton (mmolACAE/g Extract)
**Sugarcane**	93 ± 2.9	259 ± 1.00	2.34 ± 0.66	9.65 ± 0.26	13.46 ± 0.72	0.5 ± 0.03	3.61 ± 0.70
**Molasses**	196 ± 2.1	297 ± 6.00	1.91 ± 0.19	11.82 ± 3.16	18.46 ± 0.90	0.43 ± 0.04	6.20 ± 0.31

(values expressed are means ± S.D. of three measurements).
